# Patient preferences for treatment of acute bacterial skin and skin structure infections in the emergency department

**DOI:** 10.1186/s12913-018-3751-0

**Published:** 2018-12-04

**Authors:** Safa S. Almarzoky Abuhussain, Michelle A. Burak, Kelsey N. Kohman, Gabrielle Jacknin, Serina B. Tart, Athena L. V. Hobbs, Danyel K. Adams, Michael D. Nailor, Katelyn R. Keyloun, David P. Nicolau, Joseph L. Kuti

**Affiliations:** 1 Center for Anti-Infective Research and Development, Hartford Hospital, 80 Seymour Street, Hartford, CT 06102 USA; 2 Umm Al-Qura University, Makkah, Saudi Arabia; 3 Department of Pharmacy, Hartford Hospital, Hartford, CT USA; 4 Department of Pharmacy, Baylor University Medical Center, Dallas, TX USA; 5 Department of Pharmacy, University of Colorado Hospital, Aurora, CO USA; 6 Department of Pharmacy, Cape Fear Valley Health, Fayetteville, NC USA; 7 Department of Pharmacy, Baptist Memorial Hospital-Memphis, Memphis, TN USA; 8 Department of Pharmacy, Baystate Medical Center, Springfield, MA USA; 9 Allergan plc, Jersey City, NJ USA; 10 St. Joseph’s Hospital and Medical Center, Phoenix, AZ USA

**Keywords:** ABSSSI, Emergency medicine, Patient satisfaction, Antibiotic treatment, Antimicrobial stewardship

## Abstract

**Background:**

Limited research has assessed patient preferences for treatment disposition and antibiotic therapy of acute bacterial skin and skin structure infection (ABSSSI) in the emergency department (ED). Understanding patient preference for the treatment of ABSSSI may influence treatment selection and improve satisfaction.

**Methods:**

A survey was conducted across 6 US hospital EDs. Patients with ABSSSI completed a baseline survey assessing preferences for antibiotic therapy (intravenous versus oral) and treatment location. A follow-up survey was conducted within 30–40 days after ED discharge to reassess preferences and determine satisfaction with care.

**Results:**

A total of 94 patients completed both baseline and follow-up surveys. Sixty (63.8%) participants had a history of ABSSSI, and 69 (73.4%) were admitted to the hospital. Treatment at home was the most common preference reported on baseline and follow-up surveys. Patients with higher education were 82.2% less likely to prefer treatment in the hospital. Single dose intravenous therapy was the most commonly preferred antibiotic regimen on baseline and follow-up surveys (39.8 and 19.1%, respectively). Median satisfaction scores for care in the ED, hospital, home, and with overall antibiotic therapy were all 8 out of a maximum of 10.

**Conclusions:**

In these patients, the most common preference was for outpatient care and single dose intravenous antibiotics. Patient characteristics including higher education, younger age, and current employment were associated with these preferences. Opportunities exist for improving ABSSSI care and satisfaction rates by engaging patients and offering multiple treatment choices.

**Electronic supplementary material:**

The online version of this article (10.1186/s12913-018-3751-0) contains supplementary material, which is available to authorized users.

## Background

Skin and skin structure infections (SSSI) are among the most commonly encountered infections in patients presenting to the emergency department (ED) and are responsible for an increasing number of hospital admissions [1–4]. To identify those most likely to benefit from antibiotic therapy, a subset of SSSI that have lesions ≥75 cm^2^ are classified as acute bacterial skin and skin structure infections (ABSSSI) by the Food and Drug Administration (FDA) [5]. The most frequently identified cause of ABSSSIs in EDs is *Staphylococcus aureus*, particularly those strains that are methicillin-resistant (MRSA) [1, 4]. Historically, MRSA was rarely identified as a cause of skin infection, but more recent literature after 2000 attributes upwards of 60% of culture positive cases presenting to the ED to MRSA [6, 7]. Importantly, guidelines from the Infectious Diseases Society of America recommend that an ideal agent for the treatment of purulent or severe, non-purulent ABSSSI should include activity against MRSA [8]. Several oral (e.g., sulfamethoxazole-trimethoprim, doxycycline, clindamycin, etc.) and intravenous (e.g., vancomycin, linezolid, daptomycin, ceftaroline, etc.) antibiotics with activity against MRSA are available for consideration. Also included among these are the newer long-acting, intravenous lipoglycopeptides, dalbavancin and oritavancin [9, 10]. The treatment of ABSSSI in the ED typically falls into one of the following care plans: treatment and discharge from the ED, admission to an observation unit for monitoring, or admission to the hospital for management; intravenous antibiotics, a staple of the latter two treatment strategies, are often administered in the ED before a disposition is decided [1, 11]. Finally, when oral antibiotic therapy is a less desirable option (e.g., larger infections, previous failure, stable sepsis, etc), the long-acting, intravenous lipoglycopeptides allow the entire duration of therapy to be administered as a single dose in the ED, facilitating discharge home for follow-up. While each treatment plan requires individual provider assessment for clinical success and feasibility with respect to the patient’s clinical presentation, providers should also consider patient-centric plans that align with patient preferences, when appropriate.

Although ABSSSI do not confer a significant mortality burden, they are responsible for an average hospital stay of 5.0 days, $9895 (US$) in hospital costs, and a substantial economic burden on hospitals, third-party payers, and society due to productivity losses [4, 12]. Given the fiscal challenges facing hospitals in recent years, novel strategies to reduce unnecessary cost burden should be an important consideration in the ED [1, 11]. A study using US discharge data from 520 hospitals and 610,867 ABSSSI patient encounters in 2012 observed that 60% of all admissions were among patients with no life-threatening conditions and low Charlson Comorbidity Index scores (0–1) [13]. In a separate study of adults with ABSSSI presenting to 12 EDs, the need for intravenous antibiotic therapy was the only reason listed for admission in 41.5% of patients [14]. Collectively, these data suggest that there may be opportunities for clinically stable patients presenting to the ED to be treated in the outpatient setting, thereby completing treatment in a cost-saving setting versus hospital admission.

Of interest, the patient’s preference in this decision process is not well described. To our knowledge, there are currently no data on patient treatment preferences in ABSSSI, and only a few studies have attempted to address patient satisfaction and general preferences on receipt of antibiotics in the treatment of traumatic wounds and uncomplicated infections [15, 16].

## Materials and methods

The primary objective of this study was to understand patient preferences for treatment of their ABSSSI and what characteristics might influence predilections for treatment in the hospital versus at home or with intravenous versus oral antibiotics.

### Study design and participants

This was a multicenter, non-interventional, survey study conducted in 6 EDs at hospitals across the United States: Hartford Hospital (Hartford, CT), Cape Fear Valley Medical Center (Fayetteville, NC), Baylor University Medical Center at Dallas (Dallas, TX), University of Colorado Hospital (Aurora, CO), Baptist Memorial Hospital-Memphis (Memphis, TX), and Baystate Medical Center (Springfield, MA). The study methodology was reviewed and approved by each participating ED’s Institutional Review Board. Written informed consent was obtained from each participant.

Adult patients (≥ 18 years of age) who presented to the ED with SSSI were included. The presence of SSSI was confirmed by a local investigator prior to informed consent, and all consents were obtained while the patient was still in the ED. Patients were approached by the Infectious Diseases or Emergency Medicine (EM) pharmacists from each site for participation. EM providers directly caring for the patient were blinded to patient participation so as to not influence the standard of care treatment selection. The infection type was documented into one or more categories: cellulitis/erysipelas, wound infection, major cutaneous abscess, or some combination of the three. Patients were excluded if they were acutely-ill and met the Center for Medicare and Medicaid Services (CMS) definition for severe sepsis, which included one of the following: systolic blood pressure < 90 mmHg, mean arterial pressure < 60 mmHg, or serum lactate > 2.0 mmol/L (after an initial fluid challenge), if they had suspected necrotizing fasciitis or osteomyelitis, if they could not speak and read English, or if they were unable to provide written informed consent. In order to ensure adequate enrollment of patients who met the FDA definition for ABSSSI^5^, lesion size was measured at baseline, and no greater than 20% of patients with a lesion size < 75 cm^2^ were enrolled at each site. This design prevented biased enrollment of patients with smaller SSSIs that are most likely to be treated orally and discharged from the ED.

### Surveys

The study intervention consisted only of administering 2 surveys and collection of medical information prospectively from the medical record. The first survey, referred herein as the baseline survey, was administered in the ED immediately after informed consent was obtained. This written survey was completed by the participant unassisted by investigators and was a short one page (12 questions) document intended to collect data on the participants’ history of ABSSSI, their baseline preferences for treatment route (intravenous versus oral and frequency of doses), treatment location, factors that influenced their preference (Table 1), education level, and employment status.

**Table 1:** Questions assessing treatment location and antibiotic regimen satisfaction and preference in baseline and follow-up surveys

Question	Survey	Choices
Where would you prefer to receive antibiotic treatment for your current skin infection? Please choose the one best answer.	Baseline and Follow-Up	a. In the hospital for one or more nights b. In the hospital for less than one day (i.e. Emergency Department visit) c. At home d. At another healthcare setting (i.e. Infusion center or other clinic, rehab center or skilled nursing facility) e. I don’t have a preference
Please choose the best answer that completes the sentence. If given a choice, I would prefer the following antibiotic regimen to treat my current skin infection:	Baseline and Follow-Up	a. 1 to 4 pills by mouth each day for the next week or longer b. One single intravenous antibiotic dose c. One or two intravenous antibiotic doses each day for the next week or longer d. One or two intravenous antibiotic doses each day for the next week, then 1 to 4 pills by mouth each day e. I don’t have a preference
Where 1 is the most important to you and 7 is the least important, please number the following items in order of greatest importance to you with respect to antibiotic therapy for your current skin infection	Baseline only	a. Efficacy (i.e., you want it to work) b. Route of Administration (i.e., receiving either IV or oral c. Cost d. Adverse Events e. Treatment location (i.e., consider receiving care in a healthcare clinic or at home) f. Convenience of treatment (i.e., consider number of doses/days of therapy) g. Your doctor’s opinion
Where 0 is the worst experience possible and 10 is the best experience possible, what number would you use to rate your satisfaction with the care for your skin infection:	Follow-Up only	a. In the ED when you first received your care? b. At home? c. In the hospital? d. With the antibiotic treatment you received overall?
If you were treated again for a similar skin infection, would you find value if a single dose of an IV antibiotic in the ED treated your infection and prevented the need for hospitalization?	Follow-Up only	a. Definitely not b. Probably not c. Probably so d. Definitely so

The second survey, referred herein as the follow-up survey, was administered via a 15–20 min telephone call 30 to 40 days after their ED visit. Two research personnel from the Center for Anti-Infective Research and Development, Hartford Hospital, were trained to conduct this telephone survey for all participants. This 39 question survey repeated assessments of preference for route and location of ABSSSI treatment, missed time at work, engagement with ED providers, as well as measured participant satisfaction with their treatment in the ED, at home (if applicable), in the hospital (if admitted), and with antibiotic therapy in general (Table 1). Satisfaction was assessed on a scale of 0–10 with 0 representing the worst experience possible and 10 the best experience possible. A participant was considered lost to follow-up if they were unavailable to complete the follow-up survey by the end of the 40 day window.

### Medical information

All baseline demographic data were collected on a data collection tool from the medical record in the ED and during hospitalization, if admitted. Data collected included age, gender, race, Charlson Co-morbidity Index, details of current skin infection (type, size, location, presence of fever or leukocytosis), identified organism(s) on culture, location of patient prior to ED presentation (home, skilled nursing facility, etc.), ED disposition (admitted, discharged home, skilled nursing facility, etc.), antibiotic treatment details, hospital disposition (if admitted), and length of stay.

### Statistical analyses

All analyses were conducted on patients who responded to both the baseline and follow-up surveys. Patient characteristics and infection details are reported descriptively. Survey responses were evaluated descriptively with missing information or non-response removed from the denominator. Responses of “No Preference”, if available as an answer, were retained in the dataset during analyses. The two primary endpoints of the study were patient characteristics on presentation associated with preference for treatment in the hospital versus other settings, and treatment with an oral antibiotic versus other antibiotic regimens. Secondary analyses included patient characteristics associated with treatment at home versus other settings, treatment with a single intravenous dose to complete treatment versus other antibiotic regimens, and patient satisfaction scores by admission disposition. Categorical data were assessed by chi-square and continuous data were compared with t-test or Mann Whitney rank sum, as appropriate. Any variables with a *p*-value < 0.2 on univariate analysis were included in multiple logistic regression models for each preference measurement. A backward stepwise approach was used to assess the final model, with assessment of Hosmer-Lemeshow statistic and log-likelihood to determine final predictive models.Patient satisfaction scores were compared by Mann Whitney rank sum for patients who were admitted versus treated at home. Statistical significance was defined at a p-value < 0.05. All analyses were performed in SigmaPlot version 13.0 (Systat Software Inc., San Jose, CA).

## Results

### Participants

A total of 155 participants were enrolled between September 2016 and June 2017, which represented approximately 6.9% of the estimated 2230 patients who presented to these EDs during enrollment with skin infection and would have been eligible for the study based on inclusion/exclusion criteria. Each hospital participated in enrollment for an average of 6.0 ± 1.9 months. Sixty-one (39%) participants were lost to follow up and did not complete the telephone survey, leaving 94 (61%) patients for analysis. All 94 included patients met inclusion/exclusion criteria. The final numbers of patients included by site were as follows: Hartford Hospital, *n* = 24; Baylor University Medical Center Dallas, *n* = 21; University of Colorado Hospital, *n* = 19; Cape Fear Valley Medical Center, *n* = 16; Baptist Memorial Hospital – Memphis, *n* = 12; and Baystate Medical Center, *n* = 2. Baseline demographics and ABSSSI infection characteristics at ED presentation including microbiology results are provided in Table 2. The majority of participants had cellulitis alone or in combination with a cutaneous abscess, wound infection, or both. As per study design, 83% of participants met the FDA definition of ABSSSI based on a wound size ≥75 cm^2^. Participants had a median lesion size of 522 cm^2^, and 9.6 and 25.5% presented with fever and leukocytosis, respectively. A mix of Gram-positive and Gram-negative bacteria were isolated, with *Staphylococcus aureus* (30.4% MRSA) being the most common pathogen in 23 of 31 patients (74.2%). It should be noted that baseline demographics and ABSSSI infection characteristics for the evaluable participants were similar to those participants who were lost to follow-up, with the exception of minor numeric differences in age, race, and ABSSSI history (Additional file 1).

**Table 2:** Patient demographics and infection characteristics at ED presentation

Demographic/Characteristic	Number (%), unless otherwise specified (*n* = 94)
Age, years, mean ± SD	52.8 ± 15.0
Male Gender	50 (53.2)
Total Body Weight, kilograms, mean ± SD	97.6 ± 37.2
Race	
White	76 (80.9)
Black/African American	17 (18.1)
Unknown	1 (1.1)
Co-morbidities	
Diabetes (Type 1 or 2)	30 (31.9)
Peripheral Vascular Disease	8 (8.5)
Chronic Kidney Disease	13 (13.8)
Congestive Heart Failure	14 (14.9)
Coronary Artery Disease	17 (18.1)
COPD, Asthma, or Emphysema	14 (14.9)
Acute or Chronic Liver Disease	5 (5.3)
Active Malignancy	7 (7.4)
HIV/AIDS	2 (2.1)
Other Immunosuppressive disease	7 (7.4)
Charlson Comorbidity Index, median (25^th^, 75^th^ percentile)	2 (0, 4)
Education, 4 year college degree or greater	12 (12.8)
Employment, working full or part-time	40 (43)
Skin Infection Characteristics	
Cellulitis/erysipelas only	50 (53.2)
Cutaneous abscess only	1 (1.1)
Wound infection only	10 (10.6)
Cellulitis + abscess	17 (18.1)
Cellulitis + wound infection	13 (13.8)
Abscess + wound infection	1 (1.1)
Cellulitis + abscess + wound infection	2 (2.1)
Lesion Size, cm^2^, Mean ± SD	522.01 ± 806.5
Met ABSSSI definition (≥ 75 cm^2^)	78 (83)
Fever on ED Presentation	9 (9.6)
Leukocytosis on ED Presentation (Normal range: 4–12 cells/mm^3^)	24 (25.5)
History of Previous ABSSSI	60 (63.8)
Relapse/failure/readmission for an ongoing ABSSSI episode (*n* = 60)	29 (48.3)
Previously received IV antibiotics for an ABSSSI episode (*n* = 60)	38 (63.3)
Microbiology	
Patients with Culture Collected	32 (34.0)
Patients with Bacteria Isolated	31 (96.8)
Patients with Gram-positive	28 (87.5)
Patients with Gram-negative	9 (28.1)
Patients with anaerobe	3 (9.4)
Patients with multiple bacteria on culture	14 (43.8)
Number of Organisms Isolated	60
Gram-positives	43 (71.7)
*Staphylococcus aureus*	23 (38.3)
MRSA rate	7 (30.4)
β-hemolytic streptococci	12 (20)
Other	8 (13.2)
Gram-negatives (all Enterobacteriaceae)	11 (18.3)

*COPD* Chronic Obstructive Pulmonary Disorder, *HIV/AIDS,* Human Immunodeficiency Virus/Acquired Immune Deficiency Syndrome, *MRSA* Methicillin-resistant *Staphylococcus aureus*

### Disposition and antibiotic treatment

Patient disposition and antibiotic treatment details in the ED, hospital, and post-discharge are provided in Table 3. The median (25th, 75th percentile) length of stay in the ED was 7.1 (4.6, 15.0) hours, and the majority of participants (*n* = 69, 73.4%) were admitted to the hospital. Median ED length of stay was similar between patients discharged home versus those admitted [6.4 (3.3, 22.1) versus 7.4 (5.0, 14.5) hours, *p* = 0.407]. Of the 69 admitted patients, 14 (20.3%) and 8 (11.5%) had Charlson co-morbidity indices of 0 and 1, respectively, and 15 (68.2%) of these presented with no fever or leukocytosis. Median (25th, 75th percentile) hospital length of stay was 4 (2, 7) days. The majority of patients received intravenous antibiotics in the ED (88.3%) and hospital (95.7%). Less patients received outpatient parenteral antibiotic therapy (OPAT) after discharge from the ED compared with discharge from the hospital [1 (4.0%) versus 14 (24.1%), *p* = 0.031]. The median (25th, 75th percentile) total duration of antibiotics was significantly shorter for patients discharged directly from the ED versus the hospital [7 (7, 10) versus 11 (6, 15) days, *p* = 0.010].

**Table 3:** Disposition, length of stay, and antibiotic treatment details for ABSSSI treatment in the ED, hospital, and upon discharge

Disposition or Antibiotic Details	ED (*n* = 94)	Hospital (*n* = 69)	Upon Discharge (n = 94)	
			From ED (*n* = 25)	From hospital (*n* = 69)
Disposition				
Home	25 (26.6)	60 (87.0)	–	–
Admit to Hospital	69 (73.4)		–	–
Skilled nursing facility	–	9 (13.0)	–	–
LOS (hours or days), median (25^th^, 75^th^ percentile)	7.1 (4.6, 15.0)	4 (2, 7)	–	–
Antibiotics prescribed to patient				
None	7 (7.4)	1 (1.4)	–	11 (15.9)
Beta-lactams	48 (51.1)	52 (75.4)	15 (60)	36 (62.1)
TMP/SMZ	12 (12.8)	4 (5.8)	12 (48)	5 (8.6)
Tetracyclines	2 (2.1)	3 (4.3)	4 (16)	11 (19)
Vancomycin	49 (52.1)	54 (78.3)	–	5 (8.6)
Clindamycin	17 (18.1)	9 (13.0)	6 (24)	4 (6.9)
Fluoroquinolones	1 (1.1)	2 (2.9)	–	5 (8.6)
Daptomycin	–	3 (4.3)	–	2 (3.4)
Linezolid	–	2 (2.9)	–	2 (3.4)
Metronidazole	–	4 (5.8)	–	2 (3.4)
LOT (days), median (25^th^, 75^th^ percentile)	–	2 (2, 4)	7 (7, 10)	10 (7, 14)
Route of administration				
IV	74 (85.1)	51 (75.8)	1 (4)	13 (22.4)
IV plus Oral	9 (10.3)	16 (23.5)	–	1 (1.7)
Oral	4 (4.6)	1 (1.5)	24 (96)	44 (75.9)

*LOS* length of stay (hours for ED column, days for hospital column), *TMP/SMZ,* trimethoprim/sulfamethoxazole, *LOT,* length of treatment, *IV* intravenous

### Survey results

**Fig. 1:**
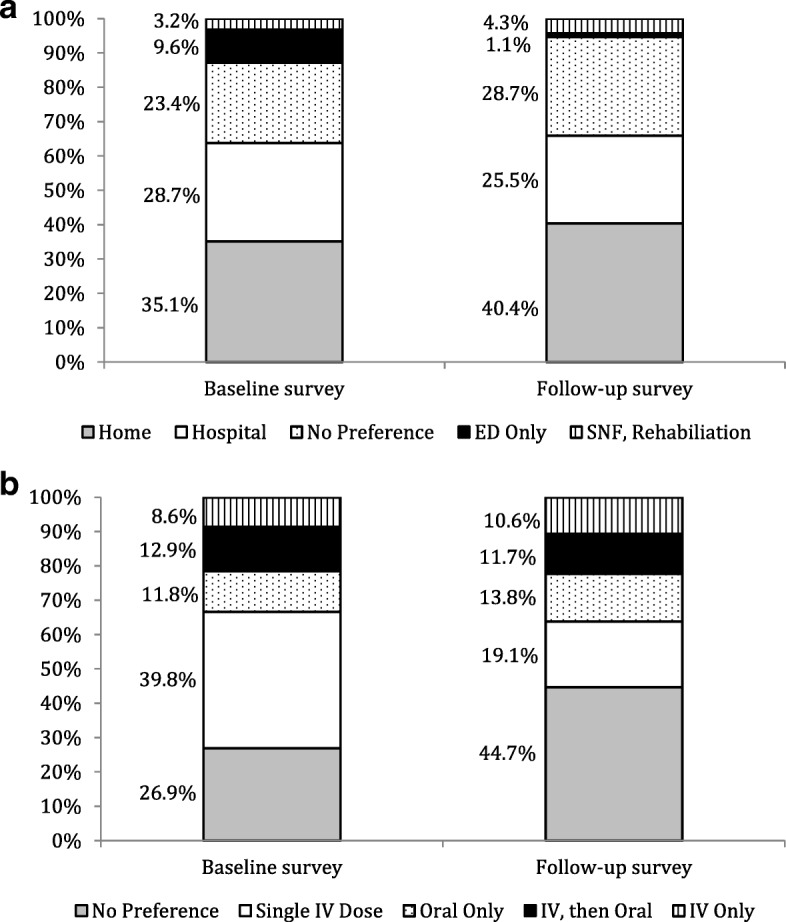
Participant’s preference selection: **a**) treatment location and **b**) antibiotic regimen on Follow-up and Baseline Surveys

Survey results for questions pertaining to preference for treatment location and treatment regimen are provided for both the baseline and follow up surveys in Fig. 1. With the exception of a single participant who did not select an antibiotic preference on the baseline survey, all 94 participants completed both surveys in their entirety. There were no differences in preference selections for treatment location between the baseline and follow-up survey (*p* = 0.23). However, a significantly greater proportion of participants preferred single dose intravenous therapy on the baseline survey compared with the follow-up survey (39.8% versus 19.1%, *p* = 0.023). The majority (*n* = 78, 83.9%) of respondents reported a definite or probable interest in single dose intravenous antibiotic therapy, if it would help avoid hospitalization. Study participants ranked the following antibiotic treatment characteristics as most to least important (median rank): Efficacy (1), Doctor’s Opinion (3), Treatment Location (4), Convenience of Treatment (4.5), Adverse Events (4.5), Route of Administration (5), and Cost (5).

Forty-three percent of participants reported they were working at the time of ED presentation, and the majority of working participants reported missing work (85%). Half (*n* = 20, 50.0%) of employed participants reported missing ≤7 days of work; 17.5% of participants missed 1–2 weeks, and another 17.5% missed more than 3 weeks. The majority of participants (*n* = 71, 75.5%) reported that the ED provider did not involve them in any decision about their care.

Median (25th, 75th percentile) satisfaction scores for care in the ED, at home, in the hospital, and with antibiotic therapy received were 8 (5, 10), 8 (6, 10), 8 (7, 10), and 8 (6, 10) out of 10, respectively. No significant differences in satisfaction scores were observed between participants who were discharged directly from the ED compared with those who were admitted to the hospital.

### Factors associated with participant preferences

Table 4 provides participant factors included in the multivariate model for treatment preference in the hospital setting. The final model included 4 year college education or greater, chronic respiratory disease, relapsed ABSSSI, and employment status at time of ABSSSI, of which only higher education was statistically significant. Patients with higher education were 82.2% less likely to prefer treatment in the hospital. Those presenting with a relapsing ABSSSI and those who were employed at baseline were also less likely to prefer hospital admission, but these factors did not achieve significance in the multivariate model. When preference for treatment at home was assessed, no variables were significant on univariate analyses (data not shown); however, 4 year college education or greater approached significance (*p* = 0.055). Preferences for treatment with oral versus intravenous antibiotics also revealed no significant patient factors (data not shown). Once again, 4 year college education approached significance in preferring oral antibiotic therapy (*p* = 0.054).

**Table 4:** Participant characteristics associated with preference for treatment in the hospital on follow-up survey

Characteristic	Univariate Analyses *p*-value*	Multivariate Analyses Odds ratio (95% Confidence Interval)	Multivariate Analyses *p*-value
COPD, Asthma, or Emphysema	0.041	2.41 (0.69–8.46)	0.170
ABSSSI relapse	0.083	0.35 (0.10–1.21)	0.097
4-year college graduate or more	0.016	0.18 (0.37–0.86)	0.032
Employed at Time of ABSSSI	0.194	0.58 (0.20–1.70)	0.322

*COPD* Chronic Obstructive Pulmonary Disorder, *HIV/AIDS* Human Immunodeficiency Virus/Acquired Immune Deficiency Syndrome

*Only variables with a p-value < 0.20 on univariate analyses were carried into multivariate logistic regression. Other variables tested included patient characteristics present on ED admission (Table 2)

Younger age, absence of diabetes, and employment at time of ABSSSI were significantly associated with preference for single dose intravenous antibiotic therapy on the baseline survey (Table 5). On the follow-up survey, only lower Charlson Co-morbidity Index was significantly associated with preference for single dose intravenous antibiotic therapy, and none of the variables were significant in multivariate analyses.

**Table 5:** Participant characteristics associated with preference for single intravenous dose to complete antibiotic therapy on baseline and follow-up surveys

Survey, Characteristic	Univariate Analyses *p*-value*	Multivariate Analyses Odds ratio (95% Confidence Interval)	Multivariate Analyses *p*-value
Baseline Survey			
Age	0.007	0.97 (0.98–1.00)	0.056
Diabetes (Type 1 or 2)	0.021	0.31 (0.11–0.89)	0.029
Employed at Time of ABSSSI	0.069	1.78 (0.682–4.63)	0.240
Follow-up Surveyǂ			
Age	0.080	0.99 (0.94–1.04)	0.678
Charlson Comorbidity Index	0.033	0.85 (0.60–1.21)	0.359
4-year college graduate or more	0.153	2.67 (0.86–8.33)	0.091

*COPD* Chronic Obstructive Pulmonary Disorder

*Only variables with a p-value < 0.20 on univariate analyses were carried into multivariate logistic regression. Other variables tested included patient characteristics present on ED admission (Table 2)

ǂ None of the identified patient characteristics were significantly associated with preference for single intravenous dose during multivariate analyses

## Discussion

SSSIs are an increasingly common reason for ED visits and hospital admissions in the last 15 years [1–4]. Given that both severity and associated mortality are low for these infections, several studies make the case that many patients can be discharged home directly from the ED without requiring further management in the hospital [4, 13, 14]. This approach could help reduce the burden of SSSI infections to the hospital as well as the patient. Of note, patient preferences for treatment have not been explored for more complicated skin infections such as ABSSSI, and such preferences may inform the decision process as well as improve patient satisfaction. This multicenter prospective study aimed at identifying patient preferences and associated factors for treatment location (hospital versus home) and antibiotic treatment (oral versus multiple dose intravenous versus single dose intravenous) after presenting to the ED with ABSSSI. To our knowledge, this is the first study of prospectively identified ABSSSI patients in the ED where preference and satisfaction were described for various healthcare treatment settings and antibiotic treatment regimens.

The National Hospital Ambulatory Medical Care Survey (NHAMCS) in 2010 reported that 4.2% of visits to the ED were caused by a SSSI [17]. These estimates were largely in agreement with the 3.4% of visits coded for a skin infection (ICD-9680–686) in our study’s EDs during the enrollment time period (data not shown). After further applying inclusion and exclusion criteria, we estimate that our study population accounts for approximately 6.9% of the eligible patients with ABSSSI. Nonetheless, the included study population appears to represent a more severely ill ABSSSI population then the most common patient presenting to an ED with a SSSI. The study eligibility criteria were designed to enroll a population that was most likely to require antibiotic therapy for their skin infection based on lesion size, yet not exclude those with history of failure or multiple infections, factors which may influence the decision to admit patients to the hospital. Indeed, we observed the majority (73.4%) of participants were admitted to the hospital. Although no reason for admission was collected in this study, observed lesion size was quite large in our population, 31.9% had diabetes, and 63.8% had a previous ABSSSI prior to the enrolling episode in this study, with nearly half presenting to the ED with a relapse or failure of an ongoing infection. This is in contrast to data by Talan and colleagues who observed admission in 15.2% of 619 patients across 12 US EDs who presented with a SSSI [14]. Notably, the lesion size was significantly smaller in that cohort; only 10.9% of patients had a lesion area ≥ 78.5 cm^2^, and only 12% had diabetes. Additionally, their patient population was slightly younger than observed in our study. Consistent with the reduction in patient/infection severity, the need for intravenous antibiotics was the sole reason for admission in 41.5% of patients in the Talan study. We have also previously observed an admission rate equal to 29.7% of all SSSI presentations (excluding necrotizing fasciitis and osteomyelitis) to one of the six EDs that participated herein [18]. In the current study, 26% of participants presented with leukocytosis and 9.6% had fever, rates that are similar to, if not slightly lower, than those for patients enrolled in recent ABSSSI trials with dalbavancin and oritavancin [9, 10]. The median (25th, 75th percentile) length of stay for admitted patients in our study was 4 (2, 7) days, which was similar to reports from the US Healthcare Cost and Utilization Project National Inpatient Sample (5 days in 2011), but shorter than observed from our hospital in our previous study (7.3 days in 2015) [4, 18].

Despite the large lesion size and frequent history of ABSSSI observed, 21.7% of admitted patients had low Charlson Co-morbidity Index scores (i.e., 0 or 1) and presented with no fever or leukocytosis. It has been suggested that patients with limited comorbidities and no acute symptoms suggestive of sepsis may be candidates for treatment in the outpatient setting [10, 13, 19]. Since many of the participants (11 of 15) with limited comorbidities had a history of a prior ABSSSI, it is possible this history in some part spurred the decision to admit. This is in line with a previous study that reported higher admission rates in patients with cellulitis recurrence [20]. Indeed, failure of prior treatment for the same skin infection was significantly associated with hospital admission in the Talan study, but was not listed as a reason for admission provided by physicians [14].

Importantly, we observed that 40% of study participants preferred to be treated at home, which was discordant with the high admission rate. Only 26% of participants in our study preferred to be treated in the hospital. These rates were similar between baseline and follow-up surveys (Fig. 1). According to Marra and colleagues, the majority of patients in an OPAT program preferred to be treated at home, and their preference was independent of their income or actual treatment location [21]. In our study, a higher education, defined by completion of a 4 year college degree or greater, was the only patient factor independently associated with a 82% lower odds of preferring hospitalization; a higher education was also numerically associated with preference for treatment at home, but did not obtain statistical significance (*p* = 0.055). Higher education levels could suggest a better understanding of health status, the goals of ABSSSI treatment, or could reflect a better home environment to provide self-care. Additionally, a higher education level could be associated with increased employment responsibilities and therefore, less opportunity for missed time at work, which was quite significant in this population (85% of employed participants reported missing work).

When surveyed on choice of antibiotic regimen, the most frequent preference was to receive a single dose intravenous antibiotic. On baseline survey, 39.8% of participants selected this as their preferred treatment regimen; on follow-up survey, however, the most common response was “no preference”. Despite this discordance, the large majority (83.9%) of participants responded with interest in a single dose intravenous antibiotic therapy on follow-up survey. No participant in this study received a long-acting lipoglycopeptide during treatment, so we assume patients were not aware such a therapy existed, and it’s important to note that some patients indeed were not interested in such a regimen, perhaps due to beliefs that multiple doses or multiple days of therapy are necessary for effectiveness. Using data collected from the baseline survey, younger age and the absence of diabetes were associated with preference for the single dose intravenous antibiotic therapy. Employment status was included in the model to control for confounding, but was not statistically significant. No patient factors were significantly associated with preference for this therapy on the follow up survey. We were also not able to identify any patient factors that were significantly associated with preference for oral or intravenous antibiotic therapy, although patients with higher education were numerically more likely to prefer oral therapy (*p* = 0.054). This observation is in agreement with their preference for treatment outside the hospital and is also congruent with patients’ rank order of antibiotic factors important to them, where route of therapy ranked lower than efficacy, doctors’ opinion, treatment location, and treatment convenience (number of doses).

Patients in this study were predominantly treated with intravenous antibiotics (i.e., beta-lactams and vancomycin) during both their ED stay and in the hospital. Given Gram-positive bacteria accounted for the majority of ABSSSI, the need for empiric broad spectrum beta-lactams with Gram-negative or anaerobic activity could be debated and is an opportunity for antimicrobial stewardship interventions in the ED [19]. Of note, patients who were discharged directly from the ED received a shorter total duration of antibiotic therapy compared with those discharged from the hospital. Also of interest was the greater frequency of patients discharged from the hospital on OPAT compared with those from the ED, which also suggests increased severity of infection in these patients, which was not otherwise captured on our data collection tool.

Despite differences in actual treatment received versus their preference, these participants were generally satisfied with their care in the ED, at home, in the hospital, and with their antibiotic regimen overall. Median satisfaction scores were 8 out of 10. Additionally, we observed no differences in satisfaction scores when comparing patients discharged directly from the ED compared with those who were admitted. While a median rank of 8 suggests good satisfaction, there is room for improvement. Currently, hospitals receive reimbursement for inpatients from the Center for Medicare and Medicaid Services (CMS) based, in part, on patient satisfaction scores [22]. Previous satisfaction studies focused around antibiotics have generally observed increased satisfaction with more informed patients [23]. However, we found that only few participants reported being involved in the treatment decisions around their ABSSSI. We hypothesize that increased communication with patients with ABSSSI in the ED and offering educated choices based on their preferences would lead to increased satisfaction, but this would require confirmation in a future prospective study.

## Limitations

Noted limitations associated with this study include the high frequency of larger lesions and the high admission rate of this population. These patients may represent a more severe infection than is routinely seen in EDs; therefore, our results may not represent the preferences of patients with more simple SSSIs. That said, 17% of participants did have smaller lesion sizes, and their preferences were not different from those with larger lesions that met the definition of ABSSSI; there was also no association with lesion size and preferences for disposition or route of antibiotic therapy in the univariate and multivariate analyses. Second, the evaluable population was small and only represented about 6.9% of eligible patients presenting with SSSI in these EDs. Third, Spanish only speaking patients were excluded due to the requirement for a follow-up survey to be conducted via telephone by non-Spanish speaking individuals. Larger studies with inclusion of patients of more varying ethnicities are needed to confirm the patient characteristics associated with certain preferences observed herein. Notably, the length of time between the baseline and follow up surveys was long at 30–40 days. This duration may have caused recall bias when responding to the follow up telephone survey, which may explain some differences in responses between both surveys. However, all follow up surveys were delivered by one of two individuals trained to address perceived participant confusion. As a part of survey study design, loss at follow-up occurs. Nonetheless, overall results from the baseline and follow-up survey were similar when compared. Additionally, the option for selecting “No Preference” on certain survey questions may have allowed some participants to opt out on providing their true preference. No clinical outcome data were collected in this study, and such responses (i.e., successful versus poor) could be a factor contributing to changes in patient preference and their overall satisfaction with care. Finally, there were not sufficient patient numbers at any single participating center to analyze the data by site to see if regional differences might affect preferences for treatment location and antibiotic choice.

## Conclusion

Our observations suggest that opportunities remain for improving treatment decision processes and satisfaction of patients presenting to the ED with ABSSSI. We identified certain patient characteristics including higher level of education, younger age, and current employment in these patients with more severe ABSSSI that were associated with preferences for treatment outside of the hospital and with route of antibiotic therapy. We conclude that ED providers should engage patients on their treatment preferences and involve them in the decisions around treatment plans. Further studies that offer treatment choices for patients presenting with ABSSSI are needed to determine if such an approach leads to improved outcomes and satisfaction.

## Additional file

Additional file 1: Patient demographics and infection characteristics at ED presentation. Contains detailed table of demographics for evaluable patients completing the study compared with those who were enrolled, but lost to follow-up. (DOCX 17 kb)

## Abbreviations

ABSSSI: Acute Bacterial Skin and Skin Structure Infections
CMS: Center for Medicare and Medicaid Services
ED: Emergency Department
FDA: Food and Drug Administration
MRSA: Methicillin-Resistant *Staphylococcus aureus*
OPAT: Outpatient Parenteral Antibiotic Therapy
SSSI: Skin and Skin Structure Infections
