# Supplementary material for: Patient preferences for treatment of acute bacterial skin and skin structure infections in the emergency department
Source: BMC Health Serv Res. 2018 Dec 4;18:932. doi: 10.1186/s12913-018-3751-0 (PMC6278032; doi:10.1186/s12913-018-3751-0)
Supplement: Supplementary file 1 — Patient demographics and infection characteristics at ED presentation. Contains detailed table of demographics for evaluable patients completing the study compared with those who were enrolled, but lost to follow-up. (DOCX 17 kb) [file 12913_2018_3751_MOESM1_ESM.docx]

**ADDITIONAL FILES**

**A1.** Patient demographics and infection characteristics at ED presentation

| **Demographic/Characteristic** | **Final Evaluable Patients**  **(n=94)** | **Enrolled Patients Lost to Follow-up**  **(n=61)** |
| --- | --- | --- |
| Age, years, mean ± SD | 52.8 ± 15.0 | 44.5 ± 14.6 |
| Male Gender | 50 (53.2) | 33 (54.0) |
| Total Body Weight, kilograms, mean ± SD | 97.6 ± 37.2 | 98.2 ± 44.7 |
| Race |  |  |
| White | 76 (80.9) | 41 (67.2) |
| Black/African American | 17 (18.1) | 17 (27.9) |
| Unknown | 1 (1.1) | 1 (1.6) |
| Co-morbidities |  |  |
| Diabetes (Type 1 or 2) | 30 (31.9) | 18 (29.5) |
| Peripheral Vascular Disease | 8 (8.5) | 4 (6.6) |
| Chronic Kidney Disease | 13 (13.8) | 8 (13.1) |
| Congestive Heart Failure | 14 (14.9) | 5 (8.2) |
| Coronary Artery Disease | 17 (18.1) | 7 (11.5) |
| COPD, Asthma, or Emphysema | 14 (14.9) | 9 (14.8) |
| Acute or Chronic Liver Disease | 5 (5.3) | - |
| Active Malignancy | 7 (7.4) | - |
| HIV/AIDS | 2 (2.1) | 2 (3.3) |
| Other Immunosuppressive disease | 7 (7.4) | 3 (4.9) |
| Charlson Comorbidity Index, median (25^th^, 75^th^ percentile) | 2 (0, 4) | 0 (0, 2) |
| Education, 4 year college degree or greater | 12 (12.8) | 12 (19.7) |
| Employment, working full or part-time | 40 (43) | 27 (45.0) |
| Skin Infection Characteristics |  |  |
| Cellulitis/erysipelas only | 50 (53.2) | 28 (45.9) |
| Cutaneous abscess only | 1 (1.1) | 5 (8.2) |
| Wound infection only | 10 (10.6) | 6 (9.8) |
| Cellulitis + abscess | 17 (18.1) | 9 (14.8) |
| Cellulitis + wound infection | 13 (13.8) | 9 (14.8) |
| Cellulitis + abscess + wound infection | 2 (2.1) | 4 (6.6) |
| Lesion Size, cm^2^, Mean ± SD | 522.01 ± 806.5 | 505.9 ± 688.8 |
| Met ABSSSI definition (≥ 75 cm^2^) | 78 (83) | 46 (75.4) |
| Fever on ED Presentation | 9 (9.6) | 6 (9.8) |
| Leukocytosis on ED Presentation (Normal range: 4 - 12 cells/mm^3^) | 24 (25.5) | 22 (36.1) |
| History of Previous ABSSSI | 60 (63.8) | 26 (42.6) |
| Relapse/failure/readmission for an ongoing ABSSSI episode | 29 (48.3)  (n=60) | 18 (69.2)  (n= 26) |
| Previously received IV antibiotics for an ABSSSI episode | 38 (63.3)  (n=60) | 22 (84.6)  (n= 26) |

*COPD,* Chronic Obstructive Pulmonary Disorder; *HIV/AIDS,* Human Immunodeficiency Virus/Acquired Immune Deficiency Syndrome; *MRSA,* Methicillin-resistant *Staphylococcus aureus*
